# Epstein-Barr virus and cytomegalovirus infections and their clinical relevance in Egyptian leukemic pediatric patients

**DOI:** 10.1186/s12985-017-0715-7

**Published:** 2017-03-06

**Authors:** Samah Aly Loutfy, Maha A. Abo-Shadi, Mohamed Fawzy, Mohamed El-Wakil, Shimaa A. Metwally, Manar M. Moneer, Nasra F. Abdel Fattah, Sara Kassem, Ahmed Elgebaly

**Affiliations:** 1 Virology and Immunology Unit, Cancer Biology Department, National Cancer Institute, Cairo University, Fom El-Khalig, Cairo, 11796 Egypt; 2 Microbiology and Immunology Department, Faculty of Pharmacy (Girls), Al Azhar University, Nasr City, Egypt; 3 Pediatric Oncology Department, National Cancer Institute, Cairo University, Cairo, Egypt; 4 Clinical Oncology Department, Faculty of Medicine, Beni-Suef University, Beni Suef, Egypt; 5 Cancer Epidemiology and Biostatistics Department, National Cancer Institute, Cairo University, Cairo, Egypt; 6 Chemistry of natural and microbial products Department Pharmaceutical Industries Division, National Research Center, Giza, Egypt; 7 Faculty of Medicine, Al Azhar University, Nasr City, Egypt; 8 Medical Research Group of Egypt, Cairo, Egypt

**Keywords:** EBV, CMV, Pediatric leukemia, PCR, Survival

## Abstract

**Background:**

Epstein-Barr virus (EBV) and human cytomegalovirus (CMV) infections are environmental risk factors affecting the outcome of cancer due to an impairment in the cell-mediated immunity. Therefore, this study aimed to detect the frequency of EBV and CMV DNA and their association with clinical characteristics and outcome of pediatric leukemic patients.

**Methods:**

Samples of 50 immunocompromised pediatric leukemic patients and 30 apparently healthy children were subjected to the amplification of EBV DNA by one version of PCR targeting the Bam H1 W region of the genomic region of EBV, and the amplification of CMV DNA by targeting the CMV UL97 genomic region by a second round PCR. All investigations were performed on WBCs and sera. Results were correlated with the clinical and laboratory characteristics of the disease, and with overall survival.

**Results:**

EBV and CMV DNA were detected in 20 and 54% of leukemic patients, respectively. Nine out of ten patients with EBV DNA (90%) were positive for CMV DNA in their sera. The presence of EBV DNA or CMV DNA was associated with neutropenia and a low total leukocyte count (TLC) (*p* = 0.02, 0.03, respectively). The presence of severe CMV disease, longer duration of febrile neutropenia, neutropenia, lymphopenia, thrombocytopenia and the presence of EBV DNA in patients’ sera were significantly associated with worse overall survival.

**Conclusion:**

The detection of CMV disease and EBV DNA is relatively common in leukemic children and is significantly associated with a decline in the overall survival.

## Background

It is now known that Cytomegalovirus (CMV) and Epstein-Barr virus (EBV) infect about 90% of the population globally, and that infection continues in a latent phase [1]. Viral infections, particularly caused by herpesviruses, were documented as important cause of morbidity and mortality in immunocompromised patients with a hematological malignancy [2, 3]. Immunosuppression conditions –like those faced in cancer patients- can lead to reactivation years later [1, 4]. Recent evidence shows that the activity of viral proteins interferes with cellular pathways controlling growth and survival, which may lead to crucial cellular transformation [5]. EBV was first identified in cultured Burkitt’s lymphoma cells 40 years ago [6], and coinfection with EBV has been linked with Hodgkin’s disease, large cell lymphoma, and chronic lymphocytic leukemia in adults [7, 8]. Therefore, the detection and quantification of these viruses help in clinical management; and prompt early treatment in order to prevent further progression in disease course [9, 10].

CMV and EBV are the most common infections in pediatric leukemic patients as a result of leukemia-associated immunosuppression status [11, 12]. There are a few reports about the role of EBV and CMV in pediatric leukemic patients. A higher exposure to herpes simplex virus types 1 and 2 (HSV-1 and 2) among acute lymphoblastic leukemia (ALL) children in Egypt has been previously reported [2]. Moreover, Lehtinen and colleagues reported a possible association between maternal EBV infection and childhood ALL [13].

Direct detection for herpesvirus infections in immunocompromised patients allows simplifying preemptive therapy through early detection of EBV replication and is considered a high positive predictive value for the related infections [14]. The current study aimed, therefore, to detect the frequency of EBV and CMV DNA and their association with the clinical characteristics and the outcome of pediatric leukemic patients.

## Methods

### Patients

This prospective study included 50 pediatric patients with leukemia diagnosed and treated at the pediatric oncology department, National Cancer Institute (NCI), Cairo University between January 2013 and December 2014. Thirty apparently healthy normal individuals with comparable age and sex were included as a control group. The Institutional Review board (IRB) of the NCI approved the protocol (IRP NO. IRP00004025). Informed consent was obtained from all participants enrolled in the study. The inclusion criteria were children till 18 years old, both sexes, suffering from acute lymphocytic leukemia (ALL) or acute myeloid leukemia (AML). All patients were subjected to detailed history, physical examination, as well as routine clinical and laboratory investigations. As for clinical assessment, the patients were checked for fever, organomegaly, mucositis, lymphadenopathy, chest infection and duration of febrile neutropenia. Main laboratory tests included; complete blood count, lactate dehydrogenase level (LDH), erythrocyte sedimentation rate (ESR), cerebrospinal fluid examination (CSF), bone marrow aspiration (BMA) and liver and renal function tests. Echocardiographic examination, immunophenotyping, karyotyping and abdominal ultrasound were also performed. Accordingly; diagnosis, disease extent, risk stratification [15], and comorbidities were determined, and correlated with the patients’ treatment schedule. The patients were followed up during the course of treatment for at least 18 months [16].

### Response and survival

Assessment of response of acute leukemia was done after induction treatment using WHO criteria. Scoring of CMV was calculated according to Plotkin scoring system [17] (Table 1). Total score of seven or more was considered severe CMV infection. Overall survival was calculated from the date of diagnosis till the date of death or last follow-up.

**Table 1:** System for scoring severity of CMV disease [17]

Manifestation	Points
Fever	1–3
Leucopenia (<4000)	1
Thrombocytopenia (<100,000)	1
Hepatitis	1–3
Pneumonia	1–3
CNS changes	1–3
Glomerulonephritis	1–3
Arthritis	2
Muscle wasting	2
Super infection	3
GI bleed	3
Death	4

### Specimen collection

Five ml of whole blood specimen was obtained from each patient and control. Serum and leukocyte samples were extracted according to Vander Bij et al.[18] and our previously published protocols [19, 20], and stored in aliquots of 100 μl saline at −20 °C until being processed for nucleic acid extraction.

### Nucleic acid extraction

Viral DNA was extracted using QIAamp viral RNA extraction kit (Qiagen, Valencia, USA). The extraction was done according to the manufacturer’s instructions. The amount of viral DNA was measured by spectrophotometry using a Nano-Drop 2000 spectrophotometer (Thermo Scientific/US, Canada) and 100 ng of DNA template was used in the PCR assays. DNA extracts were placed on ice and used immediately for PCR, then stored at −80 °C until further subsequence analysis.

### Molecular detection

1. **Molecular detection of EBV and CMV DNA:** All samples were subjected to one version PCR assay targeting nucleotide position from 32104 to 32256 bp of BamH1 W genomic region of EBV (Accession AB850660) and nucleotide position from 2692 to 2933 bp of UL 97 genomic region of CMV (Accession FJ616285.1). The amplification was conducted in a final volume of 25 μl of AmpliTaq PCR master mix (Biosystems, Barcelona, Spain) containing 0.2 μM of outer primers using the thermal cycler (Perkin-Elmer Cetus), according to previously published protocols [21, 22]. Nucleic acids of EBV VR-1492 (ATCC-USA) and CMV VR-538 (ATCC-USA) were extracted using the same extraction procedure for the samples. EBV and CMV-positive and negative (water) controls were run in each PCR assay.
2. **Detection of amplified product by agarose gel electrophoresis:** 17 μl of the PCR product was subjected to electrophoresis on a 2% agarose gel (Sigma) in Tris-acetate buffer (TAE 1X) pH 8.2, stained with 0.5 μg/ml ethidium bromide and examined under UV transillumination and photographed. The product sizes were estimated by comparing with 100 bp DNA ladder (Gendirex, Taiwan). The amplified fragments were 153 bp for EBV [21] and 240 bp for CMV [22].
3. **Sensitivity of qualitative PCR for detection of EBV and CMV DNA in clinical specimens:** Serial dilutions were prepared from CMV and EBV-positive controls and PCR assays were performed according to the previously published protocols [21, 22].
4. **Specificity of qualitative PCR for detection of EBV and CMV DNA in clinical specimens:** It was performed by testing samples positive for HSV-1 and 2, herpesvirus 6, Varicella Zoster Virus, adenovirus and CMV or EBV according to the tested virus. Then, the PCR assay was performed as mentioned before.

### Statistical analysis

Statistical analysis was done using IBM© SPSS© Statistics version 22 (IBM© Corp., Armonk, NY, USA). Numerical data were expressed as a mean and standard deviation or median and range as appropriate. Qualitative data were expressed as frequency and percentage. Chi-square test or Fisher’s exact test was used to examine the relation between qualitative variables. Survival analysis was done using Kaplan-Meier method and comparison between two survival curves was done using log-rank test. All tests were two-tailed. A *p*-value < 0.05 was considered significant.

## Results

The median age of the 50 pediatric leukemic patients was 7 years (range: 9.5 months–18 years) while that of the 30 healthy controls was 11 years (range: 4–18 years). Male to female ratio was 1.08:1 in patients, and 2:1 in controls. The two groups were comparable in age (*p* = 0.267) and sex (*p* = 0.199). The demographic, clinical, and laboratory characteristics of patients are summarized in Table 2. All patients had fever and signs of chest infections, and 70% of them had organomegaly. Laboratory (hematological and biochemical) data of the leukemia group are illustrated in Table 3. The most observed abnormal biochemical parameters are LDH and SGOT levels observed in about 94 and 30% of patients, respectively.

**Table 2:** Demographics and clinical characteristics of pediatric leukemia patients (*n* = 50)

Characteristics	N (%)
Age, median (Range)	7 years (9.5 months–18 years)
Sex	
Males	26 (52%)
Females	24 (48%)
Clinical characteristics	
Fever	50 (100%)
Organomegaly	35 (70%)
Mucositis	22 (44%)
Lymphadenopathy	7 (14%)
Chest infection	50 (100%)
Duration of Febrile neutropenia, median (range)	26.5 days (9–60)
Severe CMV disease	32 (64%)

**Table 3:** Some laboratory parameters of leukemia patients

Laboratory parameter		Leukemia group *N* = 50	
	Normal value	High risk N (%)	High risk Median (range)
1- Liver function test (LFT):			
Serum glutamic-pyruvic transaminase (SGPT)	0-41 IU/L	7 (14)	20.1 (1–317)
High risk: Level more than 41 IU/L			
Serum glutamic oxaloacetic transaminase (SGOT)	0-32 IU/L	15 (30)	23.50 (9–273)
High risk: Level more than 32 IU/L			
Total Bilirubin	0-1.2 mg/dl	1 (2)	0.45 (0.1–1.7)
High risk: Level more than 1.2 mg/dl			
2- Kidney function test (KFT):			
Creatinine	0.8-1.3 mg/dl	3 (6)	0.4 (0.1–2.2)
High risk: Level more than 1.3 mg/dl			
Uric Acid	3.4-7 mg/dl	3 (6)	3.4 (1–24)
High risk: Level more than >7 mg/dl			
Lactate dehydrogenase (LDH)	125-220 IU/L	47 (94)	907 (11–9635)
High risk: Level more than 220 IU/L			
3- Complete blood count (CBC):			
Hemoglobin concentration (Hb)	11.5-15.5 g/dl	40 (80)	7.4 (3.9–14.1)
High risk: Level less than <11.5 g/dl			
Total leukocyte count (TLC)	4500-11000 cell/cmm	11 (22)	11950 (1080–731500)
High risk: Leucopenia <4500cell/cmm			
Platelet count (Plt)	150-440 × 10^9^/l	40 (80)	39.5 (7–694)
High risk: Thrombocytopenia <150 × 10^9^/l			
Absolute neutrophile count (ANC)	2-7 × 10^9^/l	42(84)	0.13 (0–3.83)
High risk: Neutropenia <2 × 10^9^/l			
Absolute Monocyte count (AMC)	0.2-1.2 × 10^9^/l	29 (58)	0.11 (0–9.66)
High risk: Monocytopenia <0.2 × 10^9^/l			
Absolute Lymphocyte count (ALC)	1-3.5 × 10^9^/l	23 (46)	1.2 (0–143.3)
High risk: Lymphopenia <1 × 10^9^/l			

*IU/L* International unit per liter, *mg/dl* milligram per deciliter, *g/dl* gram per deciliter

### Disease characteristics and outcome of patients

Ten patients had AML and 40 had ALL; 10 of them were T-ALL. Eighty-eight percent of patients received consolidation treatment, 6% were under maintenance therapy and 6% were under salvage therapy. At the end of the study, the mortality rate was 32/50 (64%).

### Presence of EBV and CMV DNA in studied groups

The distribution of EBV and CMV DNA in serum were significantly different between leukemic and control groups (*p* = 0.048). Likewise, the distribution of both viruses in leukocytes was significantly different between leukemic and control groups (*p* < 0.001) (Table 4).

**Table 4:** Detection of herpesviruses by PCR in both sera and WBCs of the studied groups expressed as number and percentage

Group	EBV DNA	CMV DNA	Both EBV/CMV DNA	Negative for herpesviruses
Serum				
Leukemia (*n* = 50)	1 (2.0%)	18 (36.0%)	9 (18.0%)	22 (44.0%)
Control (*n* = 30)	0 (0.0%)	14 (46.7%)	0 (0.0%)	16 (53.3%)
*p* Value	=0.048			
WBCs				
Leukemia (*n* = 50)	14 (28%)	10 (20%)	9 (18%)	17 (34%)
Control (*n* = 30)	0 (0%)	12 (40%)	0 (0.0%)	18 (60%)
*p* Value	<0.001			

EBV and/or CMV frequencies were higher in sera of leukemic patients (28/50, 56%). EBV and CMV co-infection was detected in nine leukemia patients (18%) and none of the controls. The number of patients with co-infection was too small to be associated statistically with the development of severe CMV disease (*n* = 9). There was no significant association of any of the clinical and laboratory parameters with the active co-infection.

### Sensitivity and specificity of qualitative PCR assay for detection of EBV and CMV DNA

Such assays were found to be highly specific for EBV BamH1 W region and CMV UL97 as none of the other viruses tested was amplified. The sensitivity level of qualitative PCR assay was approximately 100 copies/μl for both viruses, which represents the lowest standard dilution that could be detected.

### Relation between presence of EBV and/or CMV and demographic, clinical and biochemical parameters in leukemia patients

Mucositis was more common in leukemic patients negative for herpesviruses in serum than in positive patients, with borderline statistical significance (*p* = 0.07). Neutropenia (ANC <0.13 × 10^9^/l) was more observed in patients with EBV and/ or CMV DNA in serum than in those with EBV/CMV co-infection and negative patients, with borderline significance (*p* = 0.07) (Table 5). No significant association of presence of herpesvirus DNA with other high risk clinical parameters; lymphopenia (<1.2 × 10^9^/l), thrombocytopenia (Plt < 39.5 × 10^9^/l), and low Hb level (<7.4 g/dl) (Tables 5 and 6).

**Table 5:** Relation between demographic, clinical, and biochemical findings and CMV and/or EBV as detected in serum by qualitative PCR assay in leukemic patients

	CMV orEBV alone *n* = 19	Both positive *n* = 9	Both negative *n* = 22	*p* value
Age				0.61
≤6 years (*n* = 23)	7 (30.4%)	5 (21.7%)	11 (47.8%)	
>6 years (*n* = 27)	12 (44.4%)	4 (14.8%)	11 (40.7%)	
Sex				0.53
Male (*n* = 26)	11 (42.3%)	3 (11.5%)	12 (46.2%)	
Female (*n* = 24)	8 (33.3%)	6 (25%)	10 (41.7%)	
Mucositis				0.07
Yes (*n* = 22)	7 (31.8%)	7 (31.8%)	8 (36.4%)	
No (*n* = 28)	12 (42.9%)	2 (7.1%)	14 (50.0%)	
Organomegaly				0.72
Yes (*n* = 35)	14 (40.0%)	7 (20.0%)	14 (40.0%)	
No (*n* = 15)	5 (33.3%)	2 (13.3%)	8 (53.4%)	
DFN (days)				0.75
<26.5 (*n* = 25)	8 (32%)	5 (20%)	12 (48%)	
>26.5 (*n* = 25)	11 (44%)	4 (16%)	10 (40%)	
ANC (×10^9^/L)				0.07
<0.13 (*n* = 25)	13 (52%)	2 (8%)	10 (40%)	
≥0.13 (*n* = 25)	6 (24%)	7 (28%)	12 (48%)	
Plt count (×10^9^/L)				0.27
<39.5 (*n* = 25)	7 (28%)	4 (16%)	14 (56%)	
≥39.5 (*n* = 25)	12 (48%)	5 (20%)	8 (32%)	
Hb (gm/dL)				0.65
<7.4 (*n* = 25)	10 (40%)	3 (12%)	12 (48%)	
≥7.4 (*n* = 25)	9 (36%)	6 (24%)	10 (40%)	
AMC(×10^9^/L)				0.43
<0.11 (*n* = 25)	9 (36%)	3 (12%)	13 (52%)	
≥0.11 (*n* = 25)	10 (40%)	6 (24%)	9 (36%)	
TLC(cell/mm^3^)				0.57
<11950 (*n* = 25)	11 (44%)	5 (20%)	9 (36%)	
≥11950 (*n* = 25)	8 (32%)	4 (16%)	13 (52%)	
ALC(×10^9^/L)				0.93
<1.2 (*n* = 24)	10 (41.7%)	4 (16.7%)	10 (41.7%)	
≥1.2 (*n* = 26)	9 (34.6%)	5 (19.2%)	12 (46.2%)	

Numerical factors were divided according to their median values

*DFN* Duration of febrile neutropenia, *HG* hemoglobin, *TLC* Total leukocytic count, *Plt* platelets, *ANC* Absolute neutrophilic count, *AMC* Absolute monocytic count, *ALC* Absolute lymphocytic count

**Table 6:** Relation between demographic, clinical, and biochemical findings and CMV and/or EBV as detected in leukocytes by qualitative PCR assay in leukemic patients

	EBV alone *n* = 14	CMV alone *n* = 10	Both positive *n* = 9	Both negative *n* = 17	*p* value
Age					0.45
≤6 years (*n* = 23)	8 (34.8%)	6 (26.1%)	3 (13%)	6 (26.1%)	
>6 years (*n* = 27)	6 (22.2%)	4 (14.8%)	6 (22.2%)	11 (40.7%)	
Sex					0.94
Male (*n* = 26)	7 (26.9%)	6 (23.1%)	5 (19.2%)	8 (30.8%)	
Female (*n* = 24)	7 (29.2%)	4 (16.7%)	4 (16.7%)	9 (37.5%)	
Mucositis					0.60
Yes (*n* = 22)	4 (18.2%)	5 (22.7%)	5 (22.7%)	8 (36.4%)	
No (*n* = 28)	10 (35.7%)	5 (17.9%)	4 (14.3%)	9 (32.1%)	
Organomegaly					0.61
Yes (*n* = 35)	11 (31.4%)	8 (22.9%)	6 (17.1%)	10 (28.6%)	
No (*n* = 15)	3 (20.0%))	2 (13.3%)	3 (20.0%)	7 (46.7%)	
DFN (days)					0.79
<26.5 (*n* = 25)	7 (28.0%)	4 (16.0%)	4 (16.0%)	10 (40.0%)	
>26.5 (*n* = 25)	7 (28.0%)	6 (24.0%)	5 (20.0%)	7 (28.0%)	
ANC(×10^9^/L)					0.84
<0.13 (*n* = 25)	8 (32%)	4 (16%)	5 (20%)	8 (32%)	
≥0.13 (*n* = 25)	6 (24%)	6 (24%)	4 (16%)	9 (36%)	
Plt count(×10^9^/L)					0.90
<39.5 (*n* = 25)	7 (28.0%)	6 (24.0%)	4 (16.0%)	8 (32.0%)	
≥39.5 (*n* = 25)	7 (28.0%)	4 (16.0%)	5 (20.0%)	9 (36.0%)	
Hb(gm/dL)					0.12
<7.4 (*n* = 25)	8 (32%)	5 (20%)	7 (28%)	5 (20%)	
≥7.4 (*n* = 25)	6 (24.0%)	5 (20.0%)	2 (8.0%)	12 (48.0%)	
AMC(×10^9^/L)					0.67
<0.11 (*n* = 25)	8 (32%)	6 (24%)	3 (12%)	8 (32%)	
≥0.11 (*n* = 25)	6 (24%)	4 (16%)	6 (24%)	9 (36%)	
TLC(cell/mm^3^)					0.59
<11950 (*n* = 25)	6 (24%)	6 (24%)	6 (24%)	7 (28%)	
≥11950 (*n* = 25)	8 (32%)	4 (16%)	3 (12%)	10 (40%)	
ALC(×10^9^/L)					0.79
<1.2 (*n* = 24)	8 (33.3%)	4 (16.7%)	5 (20.8%)	7 (29.2%)	
≥1.2 (*n* = 26)	6 (23.1%)	6 (23.1%)	4 (15.4%)	10 (38.5%)	

*DFN* Duration of febrile neutropenia, *HG* hemoglobin, *TLC* Total leukocytic count, *Plt* platelets, *ANC* Absolute neutrophilic count, *AMC* Absolute monocytic count, *ALC* Absolute lymphocytic count

### Relation between presence of EBV and/or CMV and overall survival in leukemia patients

**Fig. 1:**
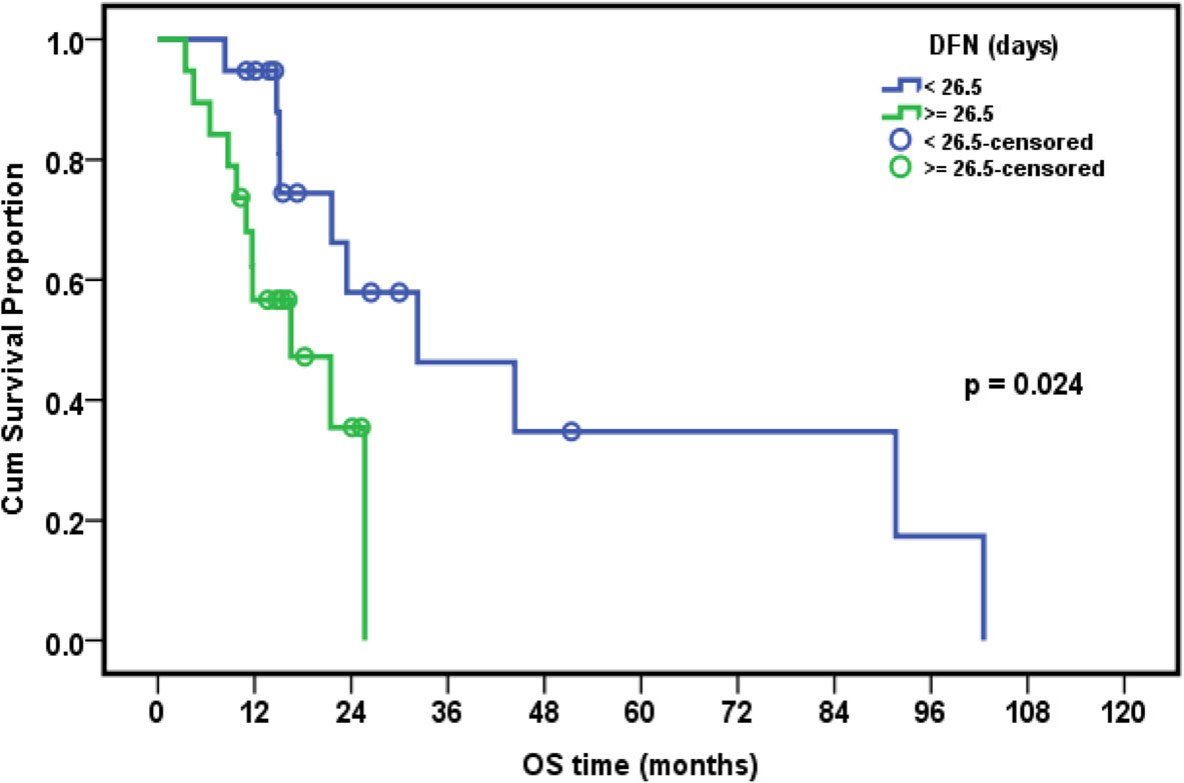
Relation of overall survival (OS) with duration of febrile neutropenia (DFN) in pediatric ALL patients

**Fig. 2:**
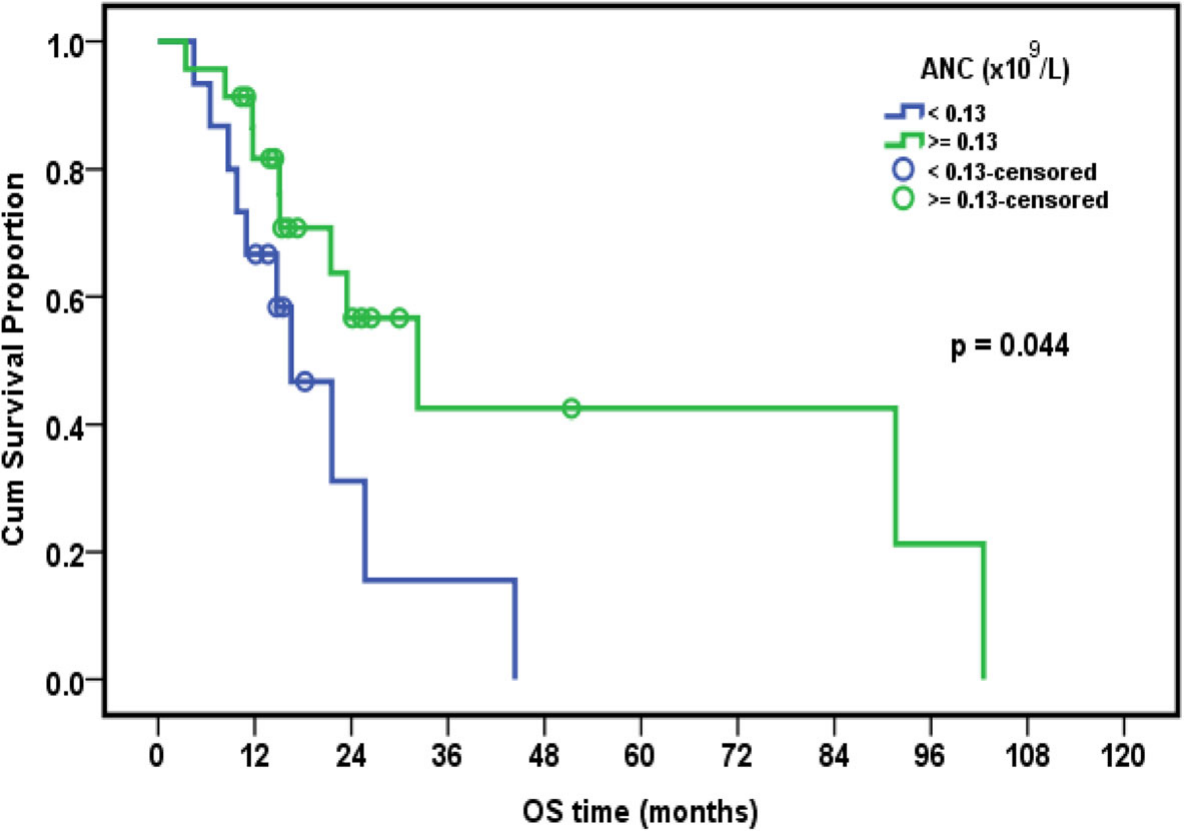
Relation of overall survival with absolute neutrophilic count (ANC) in pediatric ALL patients

**Fig. 3:**
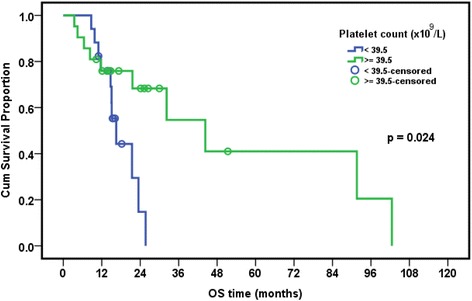
Relation of overall survival with platelet count in pediatric ALL patients

**Fig. 4:**
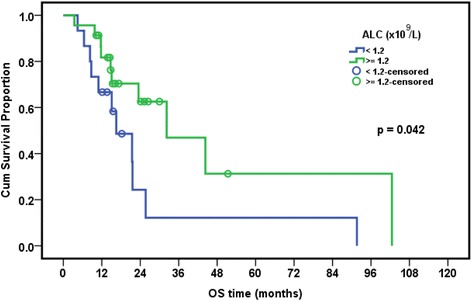
Relation of overall survival with absolute lymphocytic count in pediatric ALL patients

**Fig. 5:**
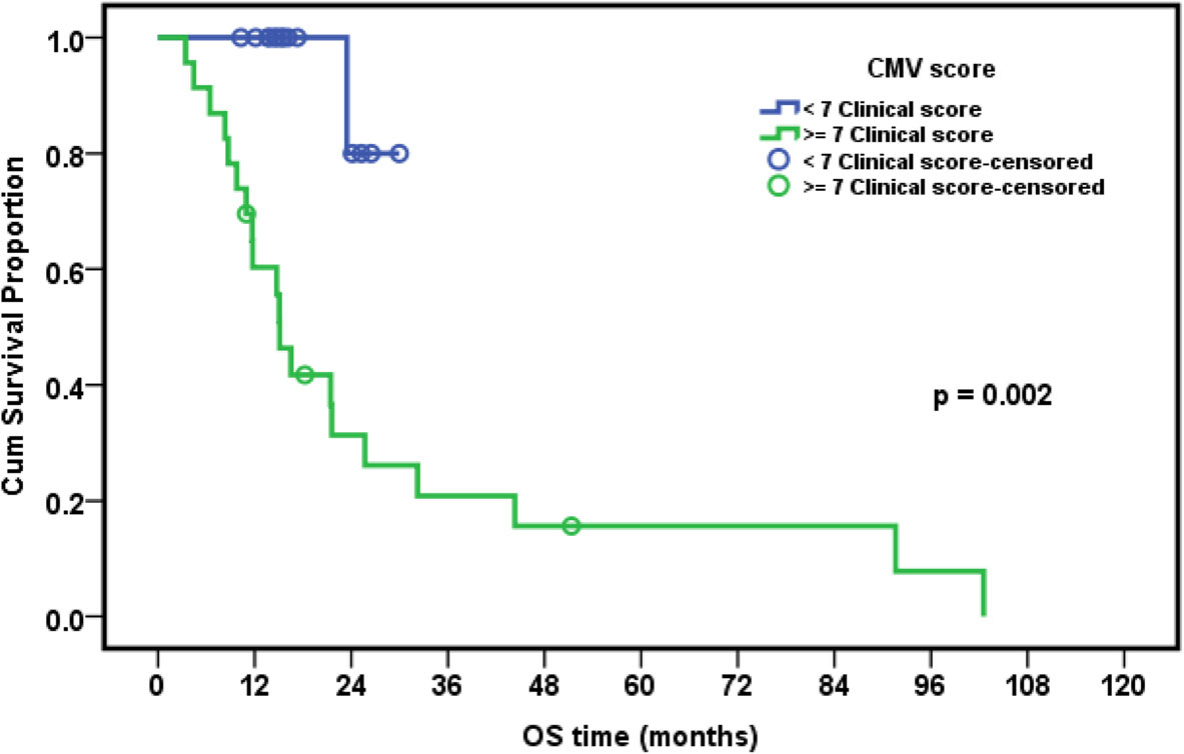
Relation of overall survival with severe CMV disease (score ≥7) in pediatric ALL patients

**Fig. 6:**
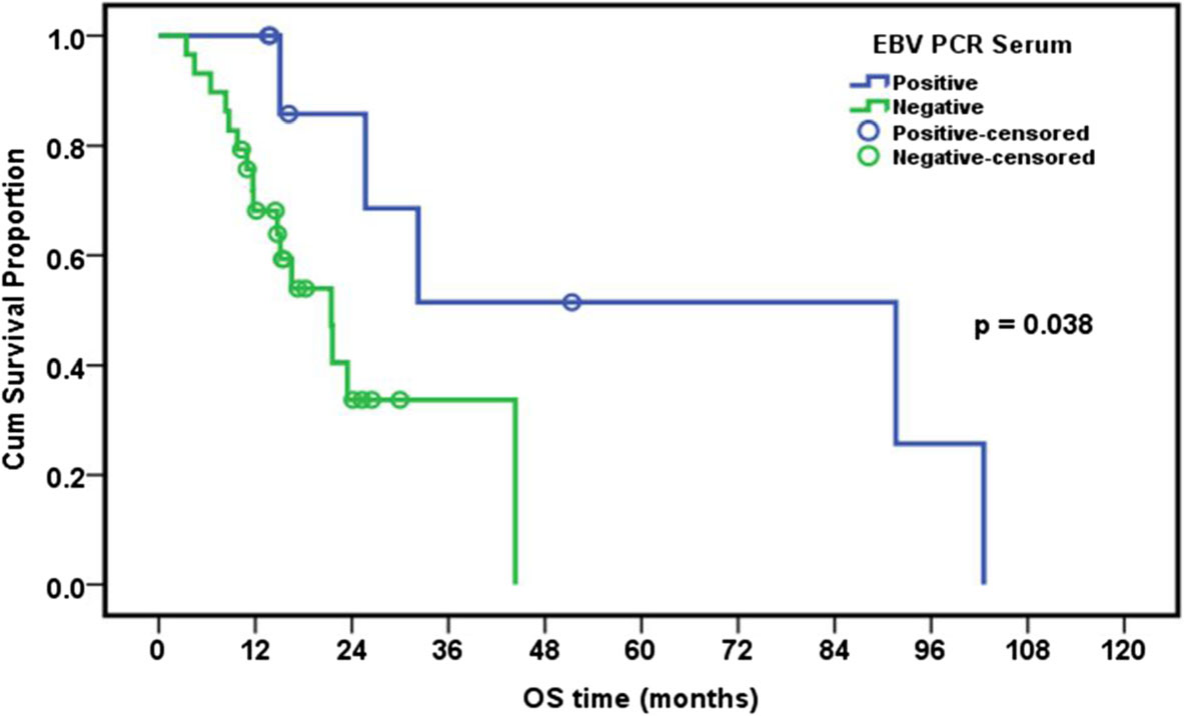
Relation of overall survival with presence of EBV DNA in serum of pediatric ALL patients

The median follow-up period of leukemia patients was 23.4 months (range: 18–28.7 months). The cumulative overall survival at 24 months was 47.4%. The Survival of pediatric leukemia patients was significantly worse in association with severe CMV disease (*p* = 0.002), longer duration of febrile neutropenia (*p* = 0.024), thrombocytopenia (*p* = 0.024), presence of active EBV infection (*p* = 0.038), lymphopenia (*p* = 0.042), and neutropenia (*p* = 0.044). EBV and CMV co-infection had borderline effect on the overall survival of patients (*p* = 0.079) (Table 7, Figs. 1, 2, 3, 4, 5 and 6).

**Table 7:** Overall survival of the leukemic patients and its relation to different prognostic factors

	Cumulative survival at 24 month (%)	Median Survival (months)	*p* value
Diagnosis			
ALL	47.4	23.5	0.518
AML	33.3	15.6	
DFN (days)			
<26.5	57.9	32.2	
>26.5	35.4	16.5	0.024
ANC (×10^9^/L)			
<0.13	15.6	16.5	
≥0.13	56.6	32.3	0.044
Plt count (×10^9^/L)			
<39.5	14.7	16.5	
≥39.5	68.3	44.3	0.024
ALC (×10^9^/L)			
<1.2	12.2	16.5	0.042
≥1.2	62.6	32.3	
CMV score			
<7 (*n* = 8)	80.0	15.1	0.002
≥7 (*n* = 20)	26.1%	23.5	
EBV PCR Serum			
Positive (*n* = 9)	68.6	91.6	
Negative (*n* = 29)	33.7	21.5	0.038
CMV&EBV in serum			
Both negative(*n* = 15)	30.8	21.6	0.079
CMV alone/EBV alone(*n* = 15)	25.9	32.3	
Both positive(*n* = 8)	62.5	91.6	

Numerical factors were divided according to their median values

*DFN* Duration of febrile neutropenia, *Plt* platelets, *ANC* Absolute neutrophilic count, *ALC* Absolute lymphocytic count

## Discussion

In recent years, a causal relationship between EBV and a variety of childhood leukemia has been demonstrated. Our previous report showed the synergistic effect of HHV6 on CMV-associated infection resulting in severe outcome among pediatric lymphoma patients [19]. This encouraged us to investigate the frequency of EBV and CMV infections and their impact when existed together on the clinical course of leukemia disease. EBV DNA whether alone or with CMV was detected in the sera of 10/50 (20%) of our leukemia patients but not in control subjects. There are few reports in literature on the role of EBV in childhood leukemia. Sehgal and coworkers have detected EBV DNA in 8/25 (32%) of pediatric ALL patients by PCR amplification of BamH1-W EBV nuclear antigen (EBNA) promoter [23]. They have also observed the presence of EBV LMP1 gene transcripts in 29/80 (36.3%) Sudanese patients with leukemia disease, but not in healthy controls (*p =* 0.0001) [24]. Moreover, It has been reported that the presence of EBV DNA in 22.8% of pediatric ALL patients, but not in the control group [25].

In the present study, CMV DNA alone or combined with EBV DNA was detected in 27/50 (54%) of the sera of leukemic patients. Debaugnies and coworkers have detected EBV and CMV DNA in 19 and 27% in the whole blood samples from immunocompromised adult and pediatric patients respectively [26].

Regarding the detection of both herpesviruses in WBCs in the present study, EBV DNA was found in 23/50 (46%) while CMV DNA in 19/50 (38%) among leukemic patients. This was higher than that reported by Lu and coworkers who have detected EBV DNA in 8/35 (22.86%) of pediatric leukemia patients using fluorescent quantitative PCR (FQ-PCR) of peripheral blood mononuclear cells [25], whereas Bonon and coworkers have reported CMV DNA in 69.5% of WBCs samples taken from adult and pediatric immunocompromised patients using nested PCR assay [27].

In the current study, CMV/EBV co-infection was found in nine patients (18%), while only one patient had a single EBV DNA detected in his serum. Zawilińska and team work have reported infections with two or three viruses dominated particularly by CMV and EBV in 65% of adult ALL-HSCT recipients’ peripheral leukocytes using nested PCR method [28]. Earlier, Drouct and colleagues had briefly reported signs of CMV and EBV co-infections in graft recipients and suggested that CMV might encourage EBV replication and dissemination [29]. Moreover, Aalto and team work have suggested that CMV may induce EBV immune reactivation following stimulation of EBV-specific memory B-cells due to iterated EBV replication [30]. EBV may require another virus like CMV to increase immunosuppressive status and mediate a suitable environment for EBV reactivation as previously reported [30, 31].

Few studies have demonstrated the impact of both EBV and/or CMV infections on the clinical course of leukemia disease. Our results revealed that no significant association of age and sex with the presence of EBV and CMV DNA Conversely, Terrazzini and his colleagues observed that effect of CMV infection depends on age and sex [32]. In the current study, the presence of EBV DNA and or CMV DNA in serum was mostly associated with mucositis and low absolute neutrophil count (ANC <1.3 × 10^9^/l) with borderline significance (*p* = 0.07). This apparently may suggest that the suppressive effect of EBV on some hematological parameters could be attributed to the presence of CMV DNA infection. However, we cannot rule out the role of leukemia treatment but could be a confounder factor that aggravated the suppressive effect of EBV or CMV. Moreover, the presence of herpesvirus-6 (HHV6) needs to be evaluated in the pediatric leukemic patients as a reactivation of HHV6 has been previously reported following treatment of Tunisian acute leukemia patients [33]. Furthermore, we have previously reported that the presence of HHV6 and CMV DNA in pediatric lymphoma patients using PCR was highly associated with more frequent episodes of febrile neutropenia, absolute neutrophil count (<0.8), lymphocytes (<0.5) and low hemoglobin level (<9.1)^18^ amongst pediatric lymphoma patients.

Severe CMV infection and active EBV infection were significantly associated with worse overall survival of leukemia patients (*p* = 0.002 and 0.038, respectively). EBV/CMV co-infection showed a borderline association with worse overall survival of patients (*p* = 0.079). These findings suggest that severe CMV infection and possibly EBV infection might play a role in the progression of leukemia and might be a useful indicator of worsening the clinical course of the disease. Similar findings were reported by Yoo and his colleagues on Korean pediatric umbilical cord blood transplant patients where the presence of CMV disease was among factors that adversely affected the survival rates of all patients [34].

## Conclusion

CMV and EBV infections are rather common in pediatric leukemic patients. Qualitative PCR assay is a hallmark for detection of both EBV and CMV DNA among leukemic patients. These infections, especially severe CMV infection, were associated with worse overall survival. Further analysis is still required to evaluate the role of HHV6 when present with EBV and CMV on woresing the immune status of pediatric leukemic patients.
